# Supplementary material for: Effects of a Digitally-Enabled Healthy Eating and Physical Activity Diabetes Prevention Peer Support Program on Weight over 6-Months
Source: Nutrients. 2025 Nov 18;17(22):3599. doi: 10.3390/nu17223599 (PMC12655732; doi:10.3390/nu17223599)
Supplement: Supplementary file 1 [file nutrients-17-03599-s001.zip › nutrients-3991277-supplementary.pdf]

Table S1: Differences in groups between program non-participants and completers

Characteristic	N	Did not complete program N = 44	Completed program N = 35	p-value
**Age**	78	56.9 (13.3)	60.6 (10.9)	0.185
**Age group**	78			0.656
Under 35 years		3 (7.0%)	1 (2.9%)	
35-44 years		13 (30.2%)	8 (22.9%)	
45-54 years		12 (27.9%)	13 (37.1%)	
55-64 years		12 (27.9%)	12 (34.3%)	
65 years and over		3 (7.0%)	1 (2.9%)	
**Gender (male)**	79	13 (29.5%)	22 (62.9%)	0.006
**Indigenous**	79	3 (6.8%)	1 (2.9%)	0.779
**Height (cm)**	68	169.3 (10.1)	169.8 (8.2)	0.812
**Weight (kg)**	70	101.2 (20.8)	95.8 (18.4)	0.257
**Waist circumference (cm)**	67	108.5 (14.5)	110.8 (15.7)	0.528
**BMI**	68	35.6 (6.1)	33.1 (6.2)	0.098
**BMI categories**	68			0.367
Normal (18.5-24.9)		1 (2.8%)	1 (3.1%)	
Overweight (25.0-29.9)		10 (27.8%)	10 (31.3%)	
Obese Class I (30.0-34.9)		11 (30.6%)	5 (15.6%)	
Obese Class II (35.0-39.9)		9 (25.0%)	6 (18.8%)	
Obese Class III (40 and above)		5 (13.9%)	10 (31.3%)	
**WHO-5 Wellbeing (1-25)**	48	13.6 (4.9)	14.9 (5.1)	0.372
**Alcohol consumption (>2 standard drinks/ day)**	79	17 (38.6%)	15 (42.9%)	0.882
**High blood fats**	79	23 (52.3%)	23 (65.7%)	0.330
**Mental health issues**	79	21 (47.7%)	10 (29.4%)	0.160
**Highest level of education**	79			0.826
Postgraduate		4 (9.1%)	4 (11.4%)	
Graduate diploma or certificate		2 (4.5%)	3 (8.6%)	
Bachelor's degree		6 (13.6%)	8 (22.9%)	
Advanced diploma or diploma		10 (22.7%)	8 (22.9%)	
Certificate		9 (20.5%)	5 (14.3%)	
Secondary school		12 (27.3%)	6 (17.1%)	
Other		1 (2.3%)	1 (2.9%)	
**Occupation**	79			0.174
Manager		4 (9.1%)	8 (22.9%)	
Professional		9 (20.5%)	6 (17.1%)	
Technician or trade worker		0 (0.0%)	1 (2.9%)	
Community or personal service worker		4 (9.1%)	1 (2.9%)	
Clerical or administrative worker		8 (18.2%)	2 (5.7%)	
Sales worker		1 (2.3%)	2 (5.7%)	
Retired		15 (34.1%)	9 (25.7%)	
Other		3 (6.8%)	6 (17.1%)	

Note. Data presented as mean (SD) or N (%); p-values calculated using two sample t-tests or Pearson's Chi-squared test.

Table S2: Between-group differences of readiness to change by gender

Characteristic	Males	Females	p-value
**Dietary behaviour – readiness to change diet**			
Do you drink water and other non-sugary drinks instead of sugary drinks/fruit juice?	23 (65.7%)	36 (81.8%)	0.169
Do you eat at least five or more servings of vegetables daily?	15 (42.9%)	16 (36.4%)	0.722
Do you eat at least two fruits every day?	18 (51.4%)	18 (40.9%)	0.481
Do you eat at least two servings of dairy foods every day?	23 (65.7%)	27 (62.8%)	0.976
Do you eat at least three different protein foods every 1-2 days?	18 (51.4%)	19 (43.2%)	0.615
Do you eat less fat overall?	16 (45.7%)	25 (56.8%)	0.451
Have you reduced amount of food you eat at each sitting?	22 (62.9%)	27 (61.4%)	1.000
Do you eat more foods with fibre?	21 (60.0%)	27 (61.4%)	1.000
Do you eat less sugary foods and carbohydrates?	21 (60.0%)	25 (56.8%)	0.956
Do you eat at regular intervals?	18 (51.4%)	28 (63.6%)	0.388
**Physical activity - readiness to change physical activity**			
Are you making yourself stronger?	10 (28.6%)	14 (32.6%)	0.894
Do you plan more activity in your day?	14 (40.0%)	16 (36.4%)	0.922
Do you plan more activity in weekends?	18 (52.9%)	15 (34.9%)	0.174
Have you increased the number of steps you take each day?	17 (48.6%)	17 (39.5%)	0.568
Have you reduced the amount of time you spend sitting?	14 (40.0%)	21 (48.8%)	0.581
**Weight – readiness to change weight**			
Are you trying to reach your best weight?	23 (65.7%)	31 (70.5%)	0.836

Note. Data presented as N (%); p-values calculated using Pearson's Chi-squared test.

Figure S1. Intention to treat mean change in weight; baseline to post-program (6 months)

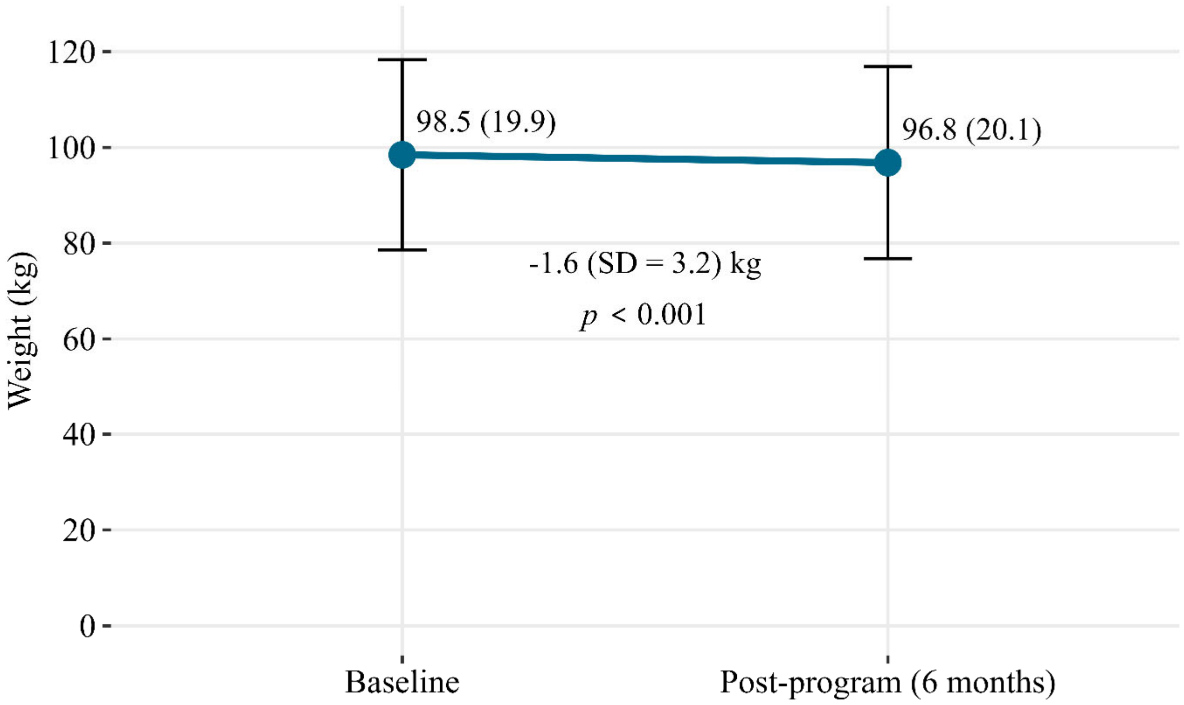

Note. Values are mean (SD). Error bars represent ±1 SD.In-figure annotation shows mean (SD) change in weight and p-value from paired t-test on change scores.
